# Optimization and antifungal efficacy against brown rot fungi of combined *Salvia rosmarinus* and *Cedrus atlantica* essential oils encapsulated in Gum Arabic

**DOI:** 10.1038/s41598-023-46858-7

**Published:** 2023-11-09

**Authors:** Saoussan Annemer, Amine Ez-zoubi, Yassine Ez zoubi, Badr Satrani, Hamid Stambouli, Amine Assouguem, Riaz Ullah, Taoufik Bouayoun, Nezha Fettoukh, Abdellah Farah

**Affiliations:** 1 Laboratory of Applied Organic Chemistry, Faculty of Sciences and Technology, University Sidi Mohammed Ben Abdellah, B P 2202, Fez, Morocco; 2 Biotechnology, Environmental Technology and Valorization of Bio-Resources Team, Department of Biology. Laboratory of Research and Development in Engineering Sciences Faculty of Sciences and Techniques Al-Hoceima, Abdelmalek Essaadi University, Tétouan, Morocco; 3 Forestry Research Center - Rabat, Avenue Omar Ibn Al Khattab, BP 763, 10050 Rabat, Morocco; 4 Forensic Sciences Institute of Royal Gendarmerie, Rabat-Institut, BP 6597, 10000 Rabat, Morocco; 5 Laboratory of Functional Ecology and Environment, Faculty of Sciences and Technology, Sidi Mohamed Ben Abdellah University, Imouzzer Street, 30000 Fez, Morocco; 6 Department of Tourism and Culinary Management, Faculty of Economics, University of Food Technologies, 4000 Plovdiv, Bulgaria; 7 Department of Pharmacognosy, College of Pharmacy, King Saud University, 4545 Riyadh, Saudi Arabia

**Keywords:** Nanobiotechnology, Nanoscale materials

## Abstract

The stability, sensitivity, and volatility of essential oils are some of their most serious limitations, and nanoencapsulation has been considered one of the most effective techniques for solving these problems. This research aimed to investigate the incorporation of *Salvia rosmarinus* Speen and *Cedrus atlantica* Manetti (MEO) essential oil mixture in Gum Arabic (GA) and to evaluate nanoencapsulation’s ability to promote antifungal activity against two brown rot fungi responsible for wood decay *Gloeophyllum trabeum* and *Poria placenta*. The optimization of encapsulation efficiency was performed using response surface methodology (RSM) with two parameters: solid-to-solid (MEO/GA ratio) and solid-to-liquid (MEO/ethanol). The recovered powder characterization was followed by various techniques using a scanning electron microscope (SEM), X-ray diffractometry (XRD), dynamic light scattering (DLS), Fourier transform infrared spectroscopy (FTIR), and thermo-gravimetric analysis (TGA). The optimal nanoencapsulating conditions obtained from RSM were ratios of MEO/GA of 1:10 (w/w) and MEO/ethanol of 10% (v/v), which provided the greatest encapsulation efficiency (87%). The results of SEM, XRD, DLS, FTIR, and TGA showed that the encapsulation of MEO using GA modified particle form and molecular structure and increased thermal stability. An antifungal activity assay indicated that an effective concentration of MEO had an inhibitory effect on brown rot fungi. It had 50% of the maximal effect (EC_50_) value of 5.15 ± 0.88 µg/mL and 12.63 ± 0.65 µg/mL for *G. trabeum* and *P. placenta*, respectively. Therefore, this product has a great potential as a natural wood preservative for sustainable construction and green building.

## Introduction

The wood industry is currently experiencing an increase in demand for a safe, and natural wood preservative^1,2^. Finding antifungal compounds in nature is crucial because of growing worries about the negative effects of commercial fungicides^3^. Researchers and the industry are paying increasingly more attention to natural anti-wood-decay fungi due to their ability to preserve the quality and safety of wood products^4^.

Essential oils are well-known viable alternatives for use in the wood preservation sector since they are non-toxic to humans and the environment^5–8^. However, the stability, water solubility, and bioavailability of the active components of essential oils are recognized as serious problems^9^, and encapsulation technology is used to overcome them^10^. The encapsulation of bioactive components using natural biopolymers has piqued researchers’ interest. Among the biopolymers, gum arabic has been extensively employed as an encapsulating material because of its stability, biodegradability, bioavailability, effectiveness, low toxicity, high-water solubility, and low solution viscosity, all of which enhance its ability to incorporate compound^11,12^.

Gum Arabic is commonly used in the textile, paint, printing, pharmaceutics, and food industries as a bulking agent, stabilizer, emulsifier, shelf-life increaser, satiating agent, and encapsulating agent for bioactive components^13^. It is a natural product of acacia exudate from the trees *Acacia senegal* and *Acacia seyal*, which belong to the Fabaceae family. This dried sap, which has a high commercial value, is a globally used product, mostly harvested in Africa and Western Asia^14^. Gum Arabic is a complex mixture of polysaccharides and proteins, composed mainly of 90% arabinogalactan, 10% arabinogalactan proteins and 1% glycoproteins^15,16^. Arabinogalactan protein is an excellent emulsifier due to its interfacial property, which ensures emulsification^17^. The arabinogalactan protein’s structure includes polypeptide chains and hydrophilic carbohydrate blocks, giving the protein favorable emulsifying property^18^.

The nanoencapsulation preparation process involves techniques such as ionic gelation, coacervation, emulsification, freeze-drying, and spray drying^19^. Freeze-drying is a commonly used method for essential oil nanoencapsulation^20^. The nanoencapsulation process is influenced by different parameters during the molecular inclusion phase. Preparation technique, volume ratio, reaction time, stirring rate, time, pH, type, the concentration of wall material, and cross-linking agents are the main parameters that can influence the complexation process^19^. Therefore, more significance should be granted to these parameters to improve nanoencapsulation and, subsequently, its application in various industrial systems.

Recently, the nanoencapsulation of essential oils in biopolymers has been widely studied in various fields, such as food preservation and pharmacology^19,21,22^. No study, however, has evaluated the potential for the nanoencapsulation of a mixture of essential oils to prevent wood against wood-decay fungi. Therefore, the current work aimed to interest in the incorporation of a *Salvia rosmarinus* and *Cedrus atlantica* essential oils mixture (MEO), which had previously been shown to exhibit optimal antifungal activity^23^, in Gum Arabic (GA) and to evaluate nanoencapsulation’s ability to promote antifungal activity against two brown rot fungi responsible for wood decay. *Cedrus atlantica*, is a significant forest tree species found in Northern Africa. This tree, also known as Atlas cedar, holds great economic and ecological importance in Morocco's Mediterranean mountains as it belongs to the Pinaceae family. Numerous studies have examined the various bio-functions of *C. atlantica*, including anti-inflammatory^24^, anticancer, antioxidant^25^, antimicrobial, insecticidal, and analgesic^26^ properties. On the other hand, rosemary, a globally renowned medicinal plant, is of paramount significance in Morocco. The essential oil derived from rosemary (previously named *Rosmarinus officinalis*) is extensively employed for the treatment of diverse ailments due to its pharmacological attributes. Multiple investigations have demonstrated that *S. rosmarinus* essential oil possesses antioxidant^27^, antimicrobial^28^, antifungal^29^, anti-inflammatory^30^, insecticidal^31^, and antiparasitic^32^ qualities. Its efficacy against fungal phytopathogens can be attributed to its high content of monoterpenes, including 1,8-cineole, camphor, and α-pinene. The nanoencapsulation condition was optimized utilizing response surface methodology (RSM), focusing on the independent parameters of the solid-to-liquid ratio of MEO/ethanol and the solid-to-solid ratio of MEO/GA and using encapsulation efficacy as dependent responses. The efficacy of this MEO-GA complex was evaluated in terms of its ability to enhance antifungal activity against brown rot fungi, including *Gloeophyllum trabeum* and *Poria placenta*.

## Material and methods

### Plant material and essential oils extraction

Leaves from *S. rosmarinus* and *C. atlantica* sawdust were collected in April from the Talsint area (Oriental region, Morocco) and the Azrou Forest (Middle Atlas Mountains, Morocco), respectively. The botanical identification was performed by Professor Satrani Badr at the Forestry Research Center Laboratory, Rabat, Morocco. The current study conformed to all applicable institutional, national, and international guidelines and regulations. Regarding collecting purposes, no specific permission is required. The MEO was extracted by simultaneous hydrodistillation utilizing a Clevenger-type apparatus as previously reported^23^. A mixture of 55% *S. rosmarinus* (110 g) and 45% *C. atlantica* (90 g), or 200 g of plants, was used. The plants were placed in two layers in the distillation flask, with *C. atlantica* wood sweats on the top and S*. rosmarinus* on the bottom*.* One liter of water was added to the distillation flask, and the mixture was boiled for 4 h. The MEO was stored at 4°C until further use.

### Chromatographic analysis

The chemical analysis of the EO mixture was performed by GC–MS (Gas chromatography coupled to mass spectrometry), and GC-FID (flame ionization detector). Component identification was accomplished using GC/MS analysis, and component quantification was performed using GC-FID analysis.

#### Gas chromatography/mass spectrometry analysis

The chemical analysis was carried out by a Hewlett-Packard (HP6890) gas chromatography coupled to a mass spectrometer (HP 5973), using a 30 m HP-5MS column ([cross-linked 5% PHME siloxane] 0.25 mm I.D., 0.25 µm film thickness). The temperature of the column was fixed at 50 °C and gradually raised to 250 °C at a rate of 2 °C/min. Helium was employed as a carrier gas, flowing at a rate of 112 mL/min and 1.5 mL/min with a split mode ratio of 1/74.7. The NIST 98 spectrum library was used to confirm the components’ MS identification. The scan mass range was between 35 and 450 m/z, the ion source temperature was 230 °C, and the ionization voltage was 70 eV.

#### Gas chromatography (GC-FID) analysis

A Hewlett-Packard gas chromatograph (HP 6890) coupled to an HP-5 capillary column was utilized to analyze the samples, an injector set to 275 °C, and an FID detector. The temperature of the oven was calibrated at 50 °C for 5 min before it was increased to 250 °C at a rate of 4 °C/min. Nitrogen was utilized as the carrier gas (1.8 mL/min).

All samples were dissolved in methanol to a ratio of 1/50, and an injection volume of 1 L was used in a split mode at a flow rate of 72.1 mL/min. Peak area normalization was utilized to determine the percentages representing the relative proportions of the EO’s components. The HP-5 MS column’s retention indices were determined using a homologous series of C_8_–C_28_ alkanes. The components were also recognized by comparing the retention indices with the retention indices mentioned in the literature.

### Encapsulation process of *S. rosmarinus* and *C. atlantica* essential oil mixture in gum arabic

**Figure 1:**
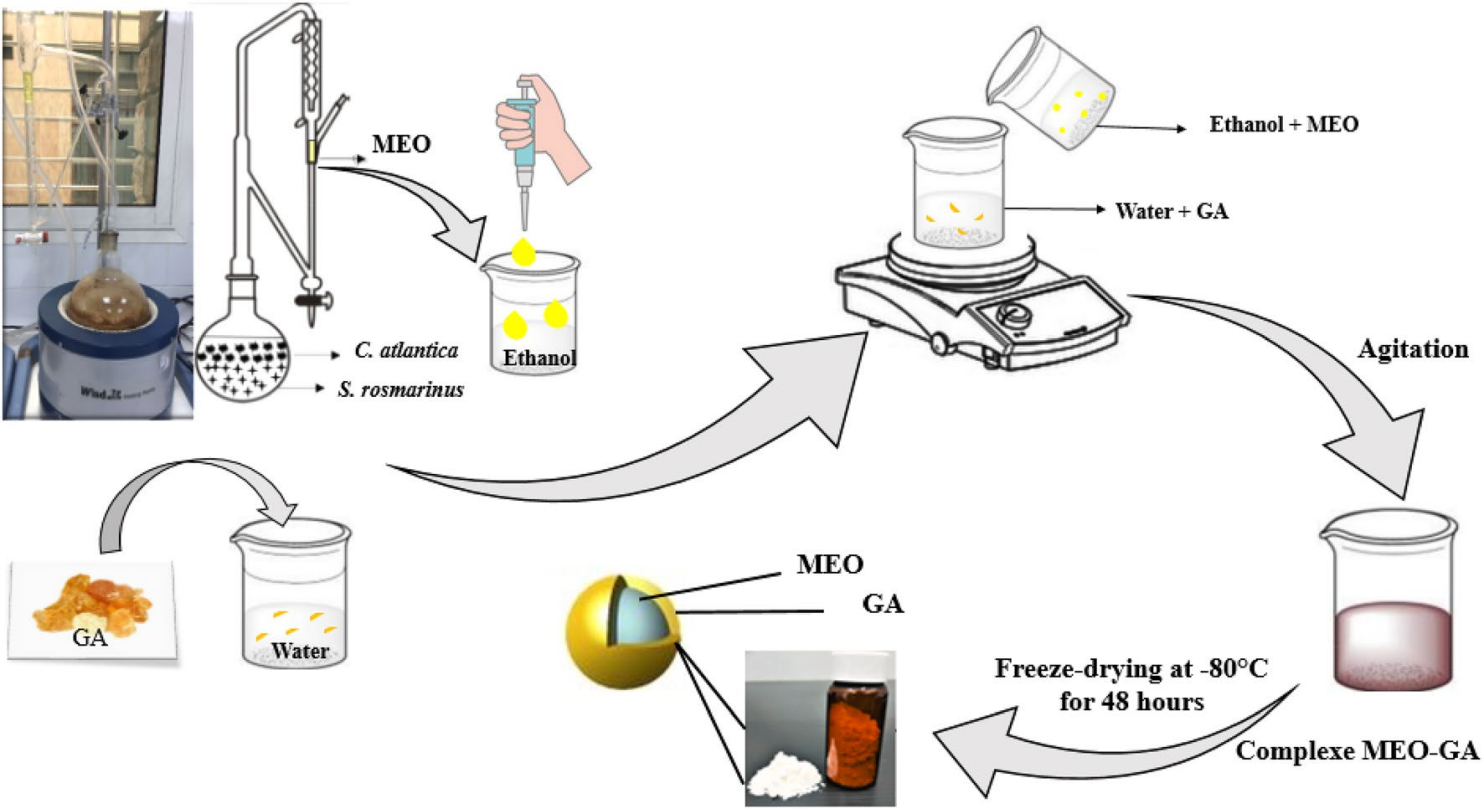
Encapsulation process illustration of MEO using GA.

The MEO-GA complexes were prepared using the freeze-drying technique described by Hu et al.^33^ A quantity of Gum Arabic (GA) was diluted in 10 mL of distilled water under a stirring magnetic field and stirred at 40 °C for 1 h, followed by filtration (0.25 µm) to eliminate the insoluble part. The MEO was then diluted in ethanol and added to the aqueous GA solution, as shown in Table 1. The mixture was then cooled to 25 °C and agitated gently in the dark. Finally, the suspension was freeze-dried at − 80 °C for 48 h according to Muhoza et al.^34^ methodology and kept at 4 °C for further analysis (Fig. 1).

**Table 1:** Two-parameter central composite design.

Runs	*X*_1_	*X*_2_
1	− 1	− 1
2	+ 1	− 1
3	− 1	+ 1
4	+ 1	+ 1
5	0	0
6	0	0
7	− 1.21	0
8	+ 1.21	0
9	0	− 1.21
10	0	+ 1.21
11	0	0
12	0	0

### Encapsulation efficiency

Encapsulation efficiency (EE) was performed based on Ez-Zoubi et al.^35^ methodology. A calibration curve was produced utilizing various EO concentrations diluted in ethanol at λ_max_ = 210 nm (BK-D580 Spectrophotometer). The sample was then diluted in ethanol and placed in an ultrasonic water bath at 50 °C, and the EE was calculated by Eq. (1).

1 $$EE \left( \% \right) = \frac{Weight\,\,of\,\, incorporated\,\,MEO}{{Total\,\,weight\,\, of\,\,MEO}} \times 100$$

### Characterization of *S. rosmarinus* and *C. atlantica* essential oil mixture in gum arabic

#### Morphology and particles size analysis

A morphological examination of the wall material (GA) and MEO inside GA (MEO/GA) was performed by scanning with an electron microscope (JSM-IT500HR), as described by Karrar et al.^36^. The samples were placed at a magnitude of 500× and a voltage of 16 kV under a high vacuum. After suspending 5 mg of MEO-GA in 20 mL of deionized water, the particle size of the sample was assessed by dynamic light scattering (Litesizer 500), following Al-Maqtari et al.^37^ methodology.

#### X-ray powder diffraction analysis

The crystallinity of MEO after the encapsulation process was achieved by X-ray diffraction (Xpert-Pro). The measurements were determined by Cu radiation to be from 5° to 50° (2θ) at room temperature, with generator settings of 40 kV and 30 mA.

#### Fourier transform infrared spectroscopy analysis

The Fourier transform infrared spectroscopy spectra (VERTEX 70 – BRUKER) of the samples above and the MEO were processed using the KBr-pellet method at a resolution of 4 cm^−1^, and 32 scans, ranging from 400 to 4000 cm^−1^, were made^38^.

#### Thermogravimetric analysis

Thermogravimetric analysis (TGA) of the samples was performed by LINSEIS STA PT1600 in an air atmosphere and heated at 10°C per minute from 20 to 600 °C, following Paula et al.^39^ methodology.

#### Antifungal activity

The antifungal activities of gum arabic (GA) and encapsulated mixture essential oil (MEO-GA) against two brown rot fungi were performed according to the Eos dispersion methodology with minor modifications^40,41^. Two brown rot fungi *Gloeophyllum trabeum* (*G. trabeum)* (Persoon ex Fries) Murril (ATCC 11539), and *Poria placenta* (*P. placenta)* (Fries) Cooke sensu J. Eriksson (ATCC 9891) were provided by the Microbiology Mycotheque Culture Collections and the Mycology Laboratory at the Forestry Research Center in Rabat, Morocco.

To conduct the experiments, the MEO and GA were serially diluted in a 0.2% sterile agar solution, and 20 mL of solid medium malt extract were added to each dilution to attain the desired concentrations (7.81, 15.63, 31.25, 62.5, 125, 250, 500, and 1000 g/L [w/v]) after the mixtures were poured into Petri dishes. The positive control (Nystatin) was prepared using the same procedure. The negative control was produced by replacing the MEO-GA and GA with a 0.2% agar solution. Each Petri dish has a 1 cm^3^ diameter piece in the center. After that, the Petri dishes were incubated for 7 days at 25 °C. Each assay was performed in triplicate. The antifungal activity of the MEO-GA and GA was assessed by determining their concentration for a 50% inhibition of mycelial growth (EC_50_) or the effective concentration for 50% of mycelium growth (EC_50_). The EC_50_ was calculated with the following equation (Eq. 2):

2 $$Mycelial\,\,growth \,\,inhibition \,\left( \% \right) = \frac{ C - T }{C} \times 100$$

where C is the average mycelial growth zone diameter (mm) for control, and T is the average mycelial growth zone diameter of the experiment (mm). Probit analysis was used to calculate the EC_50_ values (effective concentration for 50% of mycelium growth) for the studied samples (MEO-GA and GA)^42^.

### Experimental design and data analysis

**Figure 2:**
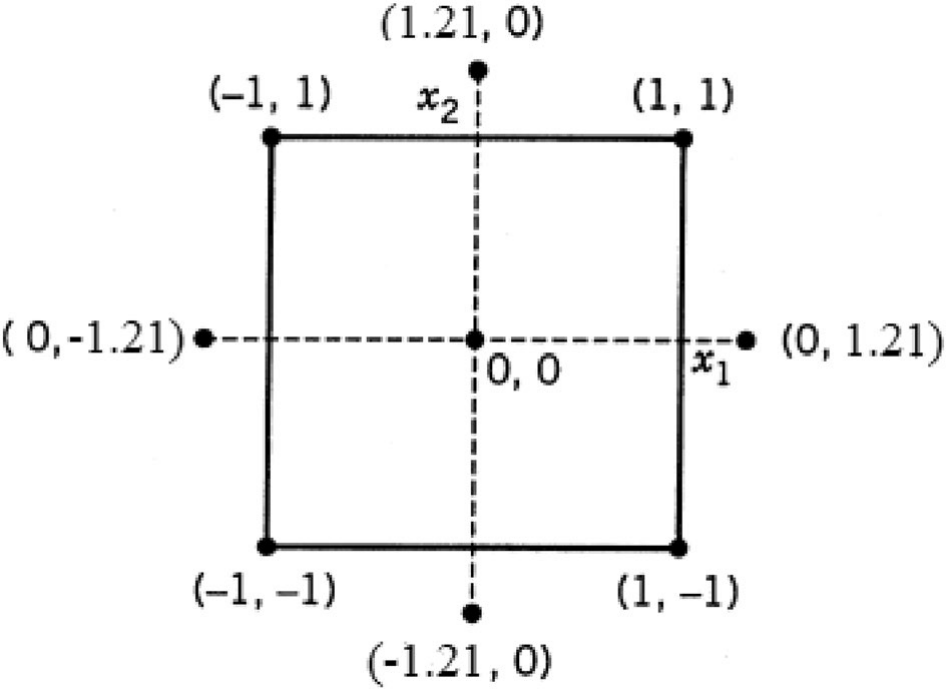
Central composite design for two parameters.

A central composite design (CCD) was conducted to investigate optimum encapsulation conditions. The effect of the two parameters (independent variables)—the solid-to-solid (MEO/GA ratio) and solid-to-liquid (MEO/ethanol ratio)—on the output response was inclusion encapsulation efficiency (*Y*), which maximized the response value. The levels of the independent parameters in the experiment were listed in Tables 1 and 2. All experiments were performed in triplicate. Notably, the design involved four runs at the square's corners, four runs in the center, and four axial runs. The corners of the square for the coded variables were (X_1_, X_2_) = (1, 1), (1, 1), (1, 1), (1, 1); the center points were (X_1_, X_2_) = (0, 0); and the axial runs were (X_1_, X_2_) = (1.21, 0), (1.21, 0), (0, 1.21). (0, 1.21) (Fig. 2).

**Table 2:** Parameters and levels utilized for central composite design.

Parameters	Code	Level-1	Level 0	Level + 1
MEO/GA (w/w)	*X*_*1*_	1:1	1:5.5	1:10
MEO/ethanol (w/v) (%)	*X*_*2*_	0	25	50

To correlate the dependent variable (*Y*) with the independent parameters (*X*_*i*_*, i* = 1, 2), a second-order multiple linear regression model was utilized as follows:

3 $$Y = a_{0} + \mathop \sum \limits_{i = 1}^{K} a_{i} X_{i} + \mathop \sum \limits_{i = 1}^{K} a_{ii} X_{ii}^{2} + \mathop \sum \limits_{i = 1}^{K} a_{ij} X_{i} X_{j} + \varepsilon$$

where *Y* is the dependent variable measured (in this study, *Y* represents encapsulation efficiency), and *a*_*0*_, *a*_*i*_, and *a*_*ii*_ indicate the regression coefficients for the constant term, second order, and interaction effects, respectively. *X*_*i*_ and *X*_*j*_ represent the coded independent studied parameters, and *k* and *ɛ* represent the total number of the optimized parameters as well as the error associated with the experiments, respectively. Multiple regressions to the fitted model were used to assess the experiments’ data. The significance of differences between the independent variables was determined using the analysis of variance test (ANOVA). The coefficients of determination (*R*^2^ and *adjusted R*^2^) were used to assess the performance of the postured models and their predictions^43,44^. The fitted model’s surface responses and contour plots were displayed using Design Expert version 13 software (Stat-Ease Society, Minneapolis, MN, USA) to visualize the relationship between the independent and dependent variables.

### Statistical analysis

Tree replicated per treatments were performed for all experiments. The means and standard deviations were determined. The significant differences among samples were examined by Tukey post hoc’s test (*p* < 0.05) utilizing OriginPro 2021 software (OriginLab, Northampton, Massachusetts, USA).

## Results and discussion

### Chemical composition

The chemical compositions of the two essential oils, *S. rosmarinus* and *C. atlantica*, alone and in combinations (55%/45%), were determined using gas chromatographic analysis and mass spectrometry (GC–MS), as shown in Table 3.

**Table 3:** Chemical composition of EO of *S. rosmarinus*, *C. atlantica* alone, and in combination.

Peak No	Compounds^a^	RI^b^	RI Lit^c^	% Relative peak area		
				*S. rosmarinus* (100%)	*S. rosmarinus: C. atlantica* (55–45%)	*C. atlantica* (100%)
1	α-Pinene	936	938	9.96 ± 2.21	6.70 ± 1.52	–
2	Camphene	952	952	3.38 ± 1.52	2.01 ± 1.97	–
3	β-Pinene	981	980	1.50 ± 1.12	0.98 ± 0.23	–
4	Myrcene	991	993	0.88 ± 0.20	0.55 ± 0.12	–
5	δ-3-Carene	1007	1011	0.08 ± 0.01	–	–
6	α-Terpinene	1019	1018	0.34 ± 0.16	0.10 ± 0.02	–
7	p-Cymene	1026	1026	2.30 ± 1.56	1.53 ± 0.12	–
8	4-Acetyl-1-methylcyclohexene	1035	1031	–	–	0.35 ± 0.02
9	1,8-Cineole	1037	1033	50.20 ± 3.80	36.25 ± 1.67	–
10	γ-Terpinene	1059	1062	0.06 ± 0.01	–	–
11	Terpinolene	1088	1089	0.21 ± 0.07	0.19 ± 0.56	–
12	Linalool	1095	1098	1.64 ± 0.43	0.97 ± 0.64	–
13	Fenchol	1116	1113	0.08 ± 0.03	–	–
14	Trans rose oxyde	1130	1127	–	–	0.92 ± 0.01
15	Camphor	1149	1144	18.47 ± 2.10	13.87 ± 1.54	–
16	Pinocarvone	1164	1163	0.31 ± 0.04	–	–
17	Borneol	1170	1166	3.56 ± 1.41	3.01 ± 1.21	–
18	Terpinen-4-ol	1179	1178	1.22 ± 0.21	0.68 ± 0.13	–
19	α-Terpineol	1184	1189	3.78 ± 1.15	0.54 ± 0.19	–
20	Myrtenal *	1192	1194	–	3.04 ± 0.25	–
21	Verbenone	1213	1205	0.54 ± 0.06	–	–
22	Carvone	1241	1243	0.15 ± 0.02	0.34 ± 0.25	–
23	Bornyl acetate	1280	1286	0.10 ± 0.01	0.08 ± 0.01	–
24	Thymol	1286	1290	0.09 ± 0.02	–	–
25	Carvacrol	1297	1293	0.19 ± 0.02	–	–
26	Longifolene	1391	1387	–	0.14 ± 0.03	0.65 ± 0.03
27	Tetradecane	1402	1399	–	–	0.82 ± 0.06
28	Himachala-2,4-diene	1409	1424	–	–	0.33 ± 0.02
29	β-Caryophyllene	1431	1419	0.38 ± 0.07	–	–
30	α-himachalene	1446	1447	–	0.20 ± 0.009	6.83 ± 1.02
31	Thujopsadiene	1463	1460	–	1.67 ± 0.87	0.51 ± 0.02
32	8,9-dehydro neoisolongifoléne	1471	1469	–	0.25 ± 0.09	0.32 ± 0.02
33	γ-Himachalene	1477	1476	–	–	3.28 ± 1.22
34	γ-Curcumene	1483	1480	–	0.79 ± 0.03	0.41 ± 0.01
35	(E)-β-Ionone	1490	1485	–	–	0.35 ± 0.02
36	β-himachalene	1499	1499	–	3.85 ± 1.14	11.23 ± 1.32
37	Cuparène	1502	1502	–	–	0.20 ± 0.09
38	α-Deshydro-ar-himachlene	1510	1511	–	0.25 ± 0.21	0.43 ± 0.01
39	δ-Cadinene	1520	1524	–	0.12 ± 0.02	0.90 ± 0.01
40	γ-Dehydro-ar-himachalene	1528	1529	–	0.32 ± 0.01	1.22 ± 1.15
41	α-Calacorene	1540	1542	–	0.28 ± 0.04	1.41 ± 1.05
42	β-Calacorene	1561	1563	–	0.97 ± 0.06	2.14 ± 0.05
43	Oxydo himachalene	1570	1574	–	–	1.51 ± 0.05
44	Turmoil	1587	1578	–	0.52 ± 0.08	1.56 ± 0.08
45	Carotol	1591	1594	–	–	0.78 ± 0.01
46	Caryophyllene oxide	1599	1581	0.08 ± 0.01	–	–
47	Cedrol	1603	1605	–	0.29 ± 0.02	1.12 ± 0.04
48	β-himachalene oxyde	1610	1611	–	1.15 ± 0.19	2.32 ± 0.07
49	Cedranone	1618	1620	–	0.34 ± 0.20	2.52 ± 1.01
50	1-Epi-cubenol	1628	1628	–	0.89 ± 0.01	2.75 ± 2.41
51	3-Iso-thujopsanone	1638	1637	–	1.44 ± 0.87	2.87 ± 2.33
52	Himachalol	1647	1647	–	2.24 ± 0.95	10.13 ± 2.04
53	Isocedranol	1661	1661	–	–	1.54 ± 1.11
54	Cadalene	1667	1674	–	0.24 ± 0.09	0.66 ± 1.45
55	β-Bisabolol	1670	1673	0.30 ± 0.02	–	–
56	Acorenone	1682	1685	0.20 ± 0.01	0.17 ± 0.01	0.94 ± 0.12
57	Deodarnone	1694	1694	–	3.54 ± 1.11	10.52 ± 2.92
58	E(E)-γ-atlantone	1707	1704	–	3.26 ± 0.61	6.14 ± 2.01
59	(Z)-α-atlantone	1719	1717	–	1.87 ± 0.45	5.32 ± 2.75
60	Khusimol	1735	1736	–	0.29 ± 0.01	1.02 ± 0.08
61	Benzyl benzoate	1763	1762	–	0.64 ± 0.06	2.48 ± 0.12
62	(E)-α-Atlantone	1783	1773	–	2.99 ± 0.71	10.33 ± 2.41
63	4-Hydroxy-muurolene	1795	1775	–	–	1.58 ± 0.12
64	14 Hydroxy-δ-cadinene	1808	1799	–	–	1.61 ± 0.08
Monoterpene hydrocarbons				18.71 ± 1.14	12.06 ± 1.13	–
Oxygenated monoterpenes				80.33 ± 3.21	58.78 ± 1.24	1.30 ± 0.16
Sesquiterpene hydrocarbons				0.38 ± 0.01	9.08 ± 1.45	31.45 ± 0.91
Oxygenated sesquiterpenes				0.58 ± 0.02	19.63 ± 1.17	67.25 ± 2.52
Total identified (%)				100 ± 0.00	99.55 ± 0.22	100 ± 0.00

^a^Components are noted in order of their elution in a HP-5 apolar column.

^b^Retention indices on an HP-5 MS column obtained experimentally using a homologous n-alkanes series (C_8_-C_28_).

^c^Retention indices obtained from Aberchane et al.^54^ and Elamrani et al.^55^;

–: Absence.

Results from triplicates are presented as mean ± standard deviation.

*New component appeared.

Twenty-six compounds were identified in the S. *rosmarinus* EO, representing a total of 100%. The Monoterpenes were predominant in *S. rosmarinus* EO (99.04% ± 0.65%). There was also a significant amount of oxygenated monoterpenes (80.33% ± 3.21%). The percentage of monoterpene hydrocarbons was 18.71% ± 1.14%, but the quantities of sesquiterpenes were almost nonexistent (0.96% ± 0.02%). The major components were 1,8-cineole (50.20% ± 3.80%), camphor (18.47% ± 2.1%), α-pinene (9.96% ± 2.21%), α-terpineol (3.78% ± 1.15%), borneol (3.56% ± 1.41%), and camphene (3.38% ± 1.52%). These compounds were similar to those found in many studies^23,45^. According to Hannour et al.^46^, *S. rosmarinus* EO from the oriental region in Morocco, the Middle Atlas Mountains, and northern Morocco are characterized by a 1,8-cineole component, whereas camphor was the most abundant compound in *S. rosmarinus* EO from Loukkos^47^.

Thirty-nine compounds were identified in the *C. atlantica* sawdust EO, representing a total of 100%. The sesquiterpenes were dominant (98.70% ± 1.72%), and there were large amounts of oxygenated sesquiterpenes (67.25% ± 2.52%) and sesquiterpene hydrocarbons (31.6% ± 0.89%). The monoterpenes were found in low percentages (1.30% ± 0.16%). The major component of the EO was β-himachalene (11.23% ± 1.32%), followed by (E)-α-atlantone (10.33% ± 2.41%), deodarnone (10.52% ± 2.92%), himachalol (10.13% ± 2.04%), α-himachalene (6.83% ± 1.02%), E(E)-γ-atlantone (6.14% ± 2.01%), (Z)-α-atlantone (5.32% ± 2.75%), and γ-himachalene (3.28% ± 1.22%). This EO was characterized by a himachalene chemotype in three isomers: α, β, and γ^48,49^. However, Zrira et al.^26^ found that α-(E)-atlantone and le β-himachalène were the most abundant components.

The chemical composition of the combination of 55% *S. rosmarinus* and 45% *C. atlantica* was determined under the same identification conditions as the previous EO. Forty-four compounds were identified, representing a total of 99.55%. Chromatography analysis showed that the 55% *S. rosmarinus* and 45% *C. atlantica* EO was mainly composed of monoterpenes (70.84% ± 1.56%), with a significant percentage of oxygenated monoterpenes (58.78% ± 1.24%) and fewer monoterpene hydrocarbons (12.06% ± 1.13%). In addition, the MEO consisted of sesquiterpene compounds (28.71% ± 1.51%) with oxygenated sesquiterpenes (19.63% ± 1.17%) and a small percentage of sesquiterpene hydrocarbons (9.08% ± 1.45%). The amount of monoterpene was higher than that of sesquiterpenes, which may be because the quantity of *S. rosmarinus* was higher than that of *C. atlantica* in the binary combination (55% of *S. rosmarinus* and 45% of *C. atlantica*). The EO composed of 55% *S. rosmarinus* and 45% *C. atlantica* mainly comprised 1,8-cineole (36.25% ± 1.67%), camphor (13.87% ± 1.54%), α-pinene (6.70% ± 1.52%), myrtenal (3.04% ± 0.25%), β-himachalene (3.85% ± 1.14%), and borneol (3.01% ± 1.21%). These compounds were detected in small amounts in binary combination, even though they were presented in the individual oil in a relatively high percentage. Simultaneous hydrodistillation led to a new component in the binary mixtures: a myrtenal compound (3.04% ± 0.25%). This finding can be explained by the interaction between the different compounds, the effect of the temperature during simultaneous hydrodistillation, or the appearance of compounds that already existed in trace amounts in the individual oils.

Results of the binary mixture's chemical composition indicated that the main compounds were nearly the same as those identified in previous studies^23^. Yet they differed in percentages due to the number of plants used in the binary combination during simultaneous hydrodistillation. In accordance with our study, Kharraf et al.^50^ found that the major components detected in EO mixtures were the same as those presented in the individual EOs. However, Muturi et al.^51^ findings differed from ours in that the number of components in the EO mixtures were lower than that in individual EOs. A new component in the chemical composition of EOs was found when mixing *A. sativum* (bulbs) and *C. paradisi* (leaves)^52^. Wangrawa et al.^53^ found that components such as isopiperitone, p-cymene, b-elemol, carvacrol, and caryophylenne were presented in the *Lippia multiflora* + *Cymbopogon schoenanthus* combination.

### Optimization of *S. rosmarinus* and *C. atlantica* essential oil mixture encapsulation condition in gum arabic

To select the parameters that most impacted encapsulation efficiency, preliminary studies were performed based on literature data to optimize the encapsulation procedure in GA^56–58^. The parameters were a solid-to-solid ratio of MEO/GA and a solid-to-liquid ratio of MEO/ethanol. Due to the low water solubility of EOs, ethanol was required as a co-solvent in the encapsulation process^59^. An ethanol concentration of greater than 50%, though, leads to low encapsulation efficiency^60^. To minimize dissipation during the inclusion process, the ratios of MEO/ethanol and MEO/GA were varied in the range of 5.8–54.2% (v/v) and 1/0.05–1/10.945, respectively^33,56^.

A central composite design (CCD) was performed to optimize the encapsulation parameters. By employing CCD, a matrix of 12 experiments was generated. Table 4 shows the observed response values for each experiment’s results.

**Table 4:** Central composite design and response variables for the preparation of the encapsulation of an EO mixture of 55% *S. rosmarinus* and 45% *C. atlantica*.

Run	Parameters		Response variables
	*X*_*1*_: MEO/GA	*X*_*2*_: MEO/ethanol (%)	Encapsulation effieciency (%)
1	1/1	10	49.00 ± 1.52
2	1/10	10	87.00 ± 3.41
3	1/1	50	55.00 ± 2.21
4	1/10	50	70.20 ± 3.52
5	1/5.5	30	67.80 ± 1.84
6	1/5.5	30	68.20 ± 1.96
7	1/0.05	30	45.20 ± 2.01
8	1/10.945	30	77.60 ± 3.24
9	1/5.5	5.8	72.00 ± 2.46
10	1/5.5	54.2	66.00 ± 1.75
11	1/5.5	30	68.00 ± 1.63
12	1/5.5	30	67.60 ± 1.44

The results of an ANOVA test conducted to validate the regression models were presented in Table 5. The results revealed that the quadratic polynomial model was a good target response. Based on these results, the probability of risk significance *p* values for the postulated model was less than 0.05, showing that the adopted model was highly significant. The probability of a lack of fit for encapsulation efficiency was 0.06, indicating that the obtained model’s lack of fit was insignificant. The encapsulation efficiency model was judged as fair for explaining the experiments’ results based on the analysis of variance and the lack of fit of the results. Furthermore, the coefficient of determination and the coefficient of determination-adjusted values (*R*^*2*^ = 0.99 and *R*_*ajd*_^2^ = 0.99) were high, demonstrating a good relationship among the observed and predicted values of the adopted model.

**Table 5:** Analysis of variance for the postulated model.

Source	Degrees of freedom	Sum of square	Mean square	*F-Ratio*	*p-value*
Model	5	1484.39	296.88	950.19	< 0.0001*
Error	6	1.87	0.31		
Total	11	1486.27			
Lack of fit	3	1.67	0.56	8.37	0.06
Pure Error	3	0.20	0.07		
*R*^2^	99.87%				
*R*_*adj*_	99.77%				
standard deviation	0.56				

*Statistically significant at *p* < 0.05.

**Figure 3:**
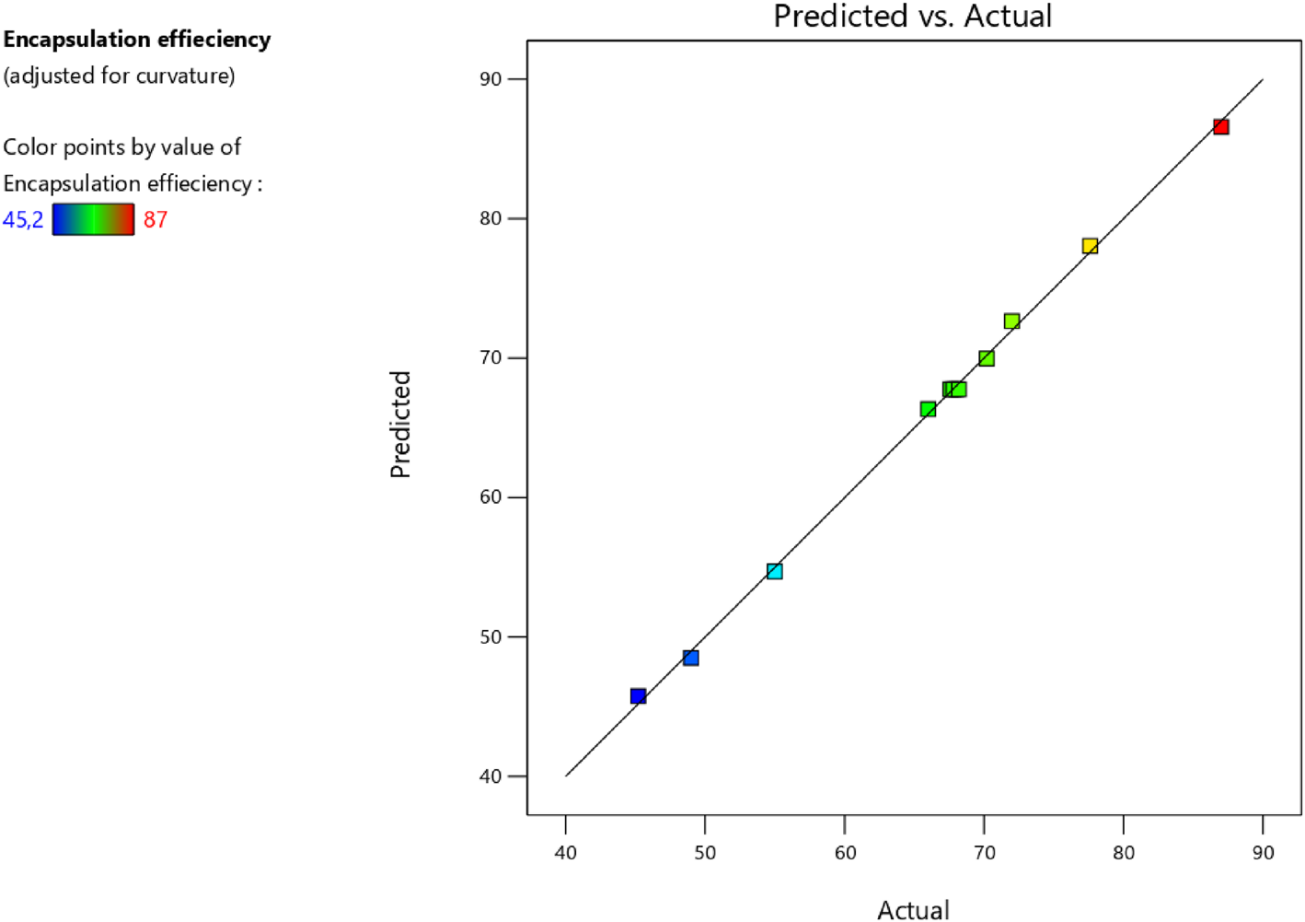
Observed vs predicted values for encapsulation efficiency.

The observed value of the output response according to the predicted value was illustrated in Fig. 3. As illustrated, most points formed a straight line, demonstrating good agreement between the experiments’ values for encapsulation efficiency and the predicted ones. This finding is consistent with the results shown in Table 5. When *R*^2^ was close to 1, the predicted points formed one line as a function of observed values^61,62^.

The significance of the coefficients in the second-order multiple linear regression models with a 95% confidence interval was shown in Table 6, which was calculated by a Student’s t-test. The least squares method was used to estimate the regression coefficients for linear, quadratic, and interaction (Table 6). All the coefficients had a highly significant effect, as the *p*-value was less than 0.05. Furthermore, it has been shown that the lower the *p*-value, the higher significant the coefficients are in the regression model^63,64^.

**Table 6:** Estimated regression coefficients and their significance in the second-order multiple linear regression models for the fitted model.

Term	Estimation	Error standard	*t ratio*	*Prob.* >*|t|*
Constant	67.77	0.27	248.08	< 0.0001*
MEO/GA	13.34	0.21	62.81	< 0.0001*
MEO/ethanol	− 2.61	0.21	− 12.28	< 0.0001*
MEO/GA*MEO/ethanol	− 5.70	0.28	− 20.39	< 0.0001*
(MEO/GA)^2^	− 4.01	0.27	− 14.86	< 0.0001*
(MEO/ethanol)^2^	1.18	0.27	4.37	0.0047*

*Statistically significant at *p* < 0.05.

The results also indicated that the concentration of the ratio of MEO/GA had the most significant impact on the EE%, followed by the interaction of MEO/GA and MEO/ethanol and the second-order interaction of MEO/GA. Thus, MEO/GA positively impacted the EE%, with a high positive t-ratio (62.81). However, the values of MEO/GA*MEO/ethanol, *(MEO/GA)*^2^, and *MEO/ethanol* had a significant negative impact on the EE%, with t ratios of − 20.39, − 14.86, and − 12.28, respectively.

The mathematical models of the response surfaces of the adopted model were presented as second-order polynomials. The equations displayed the significant terms of the model for encapsulation efficiency (EE%).

4 $$\begin{aligned} EE \% \, = & \,67.77 + 13.34 \left( {MEO{/}GA} \right) - 2.61\left( {MEO{/}ethanol} \right) \\ & \, - \,5.70 \left( ({MEO/GA)*(MEO{/}ethanol)} \right) - 4.01\left( {MEO/GA } \right)^{2} + 1.18\left( {MEO{/}ethanol } \right)^{2} + \varepsilon \\ \end{aligned}$$

**Figure 4:**
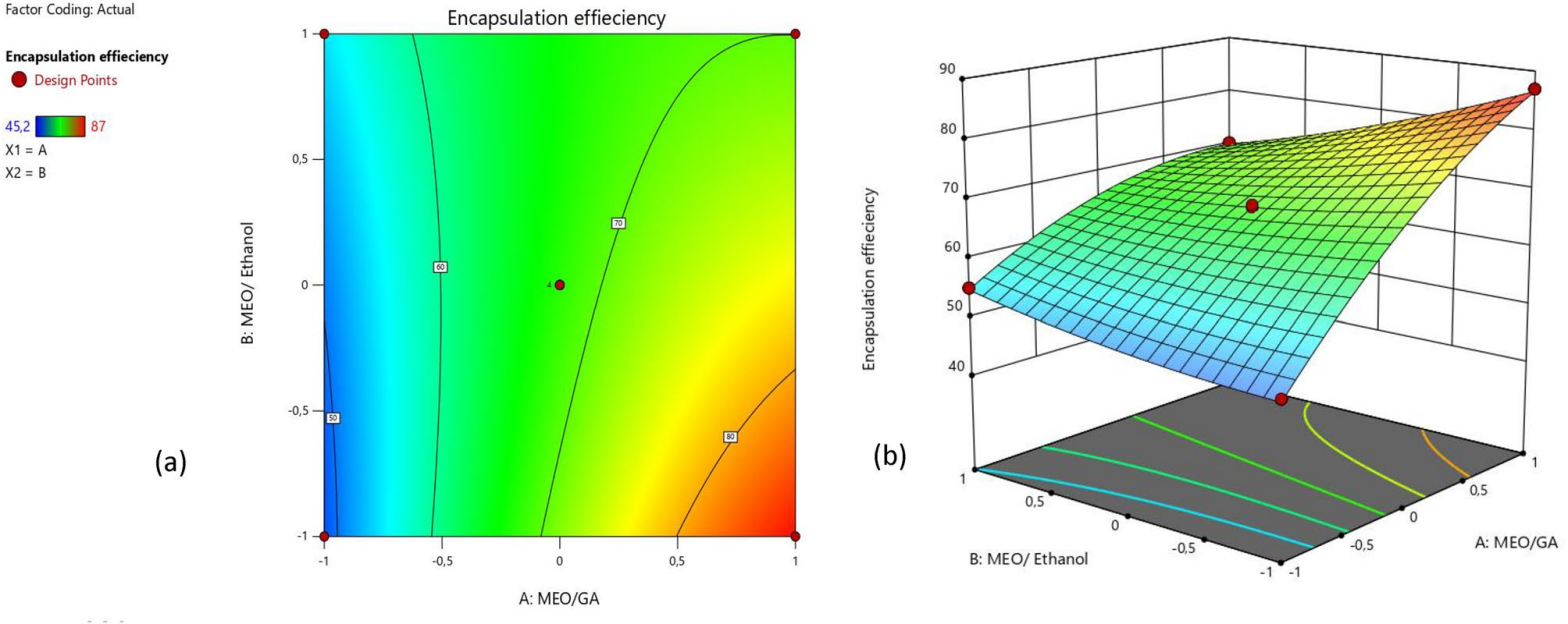
2D (**a**) and 3D (**b**) representation of the optimized parameters leading to the desired encapsulation efficiency.

After validating the models obtained from the RSM, the optimal fit of the two parameters that enabled high encapsulation efficiency was the focus of this study. The effects of the ratios MEO/GA and MEO/ethanol on encapsulation efficiency were illustrated in the response surface shown in Fig. 4. The optimum zone for the two factors exists on the area of the cube formed by the area where the ratio of MEO/ethanol is minimal and the ratio of MEO/GA is maximal.

**Figure 5:**
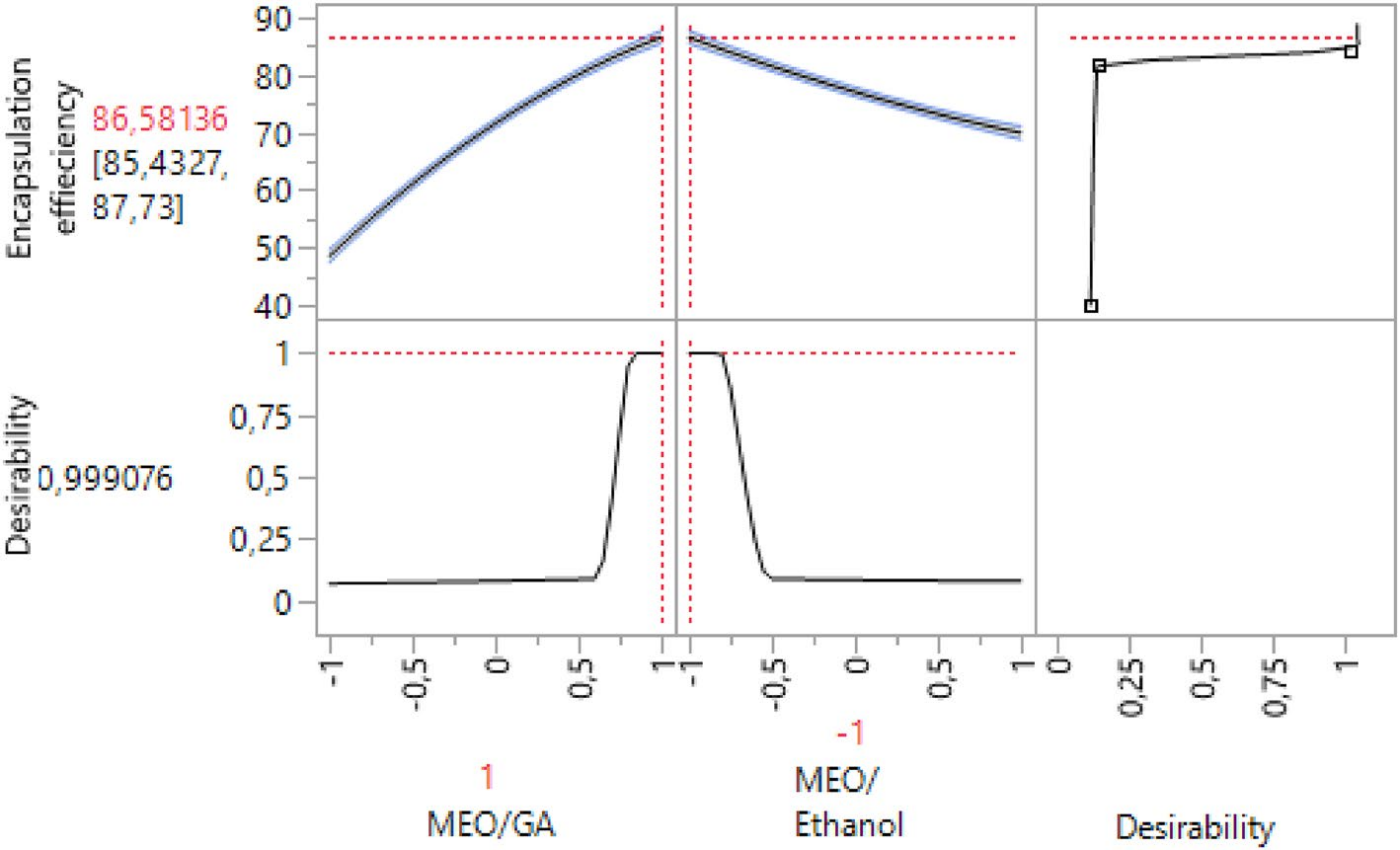
Profile for predicting optimal conditions for encapsulation efficiency.

The desirability plots corresponding to the responses for encapsulation efficiency were displayed in Fig. 5. Apparently, the encapsulation efficiency reached its maximal value (86.58%) with an MEO/GA ratio of 1/10 and an MEO/ethanol percentage of 10%, with a desirability of 99%.

The results indicated that the optimized conditions corresponding to the experimental design corner (run 2) had an MEO/ethanol volume ratio of 1:10 and an MEO/GA percentage of 10%.

This finding confirmed previous research, which indicated that encapsulation efficiency was reduced by enhancing the co-solvent ratios of ethanol^65–68^. This reduction could be explained by the high quantities of ethanol disturbing the non-covalent bonding required for essential oil (EO) incorporation in the GA. The introduction of GA improved the encapsulating ability of the MEOs. When the weighted EO-to-GA ratio was 1:10, the maximum encapsulation efficiency was 87%. The MOE emulsion droplets likely moved more freely in the medium^69^. Due to the increased amount of GA, the number of essential oil molecules diffused into the combined solution likely increased. This increase might also allow for better protection of the oil from coalescence, which eventually integrated into the GA.

### Characterization of nanocapsules

#### Morphology and particles size analysis

**Figure 6:**
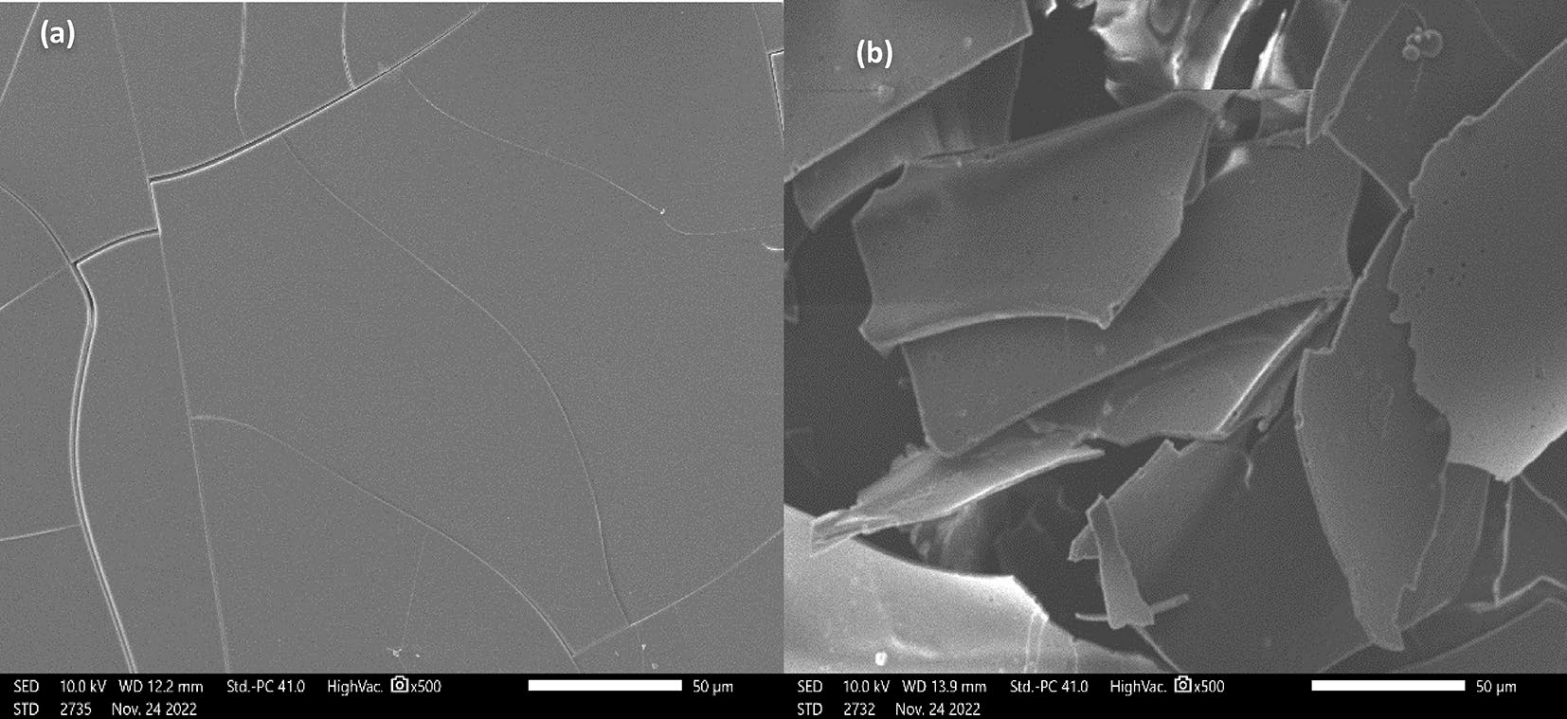
The SEM micrographs of GA (**a**) and MEO-GA (**b**) at × 500.

A scanning electron microscope (SEM) was used to study GA and MEO-GA morphology at 500×. According to Fig. 6, the pure sample of GA occurs in amorphous, irregular crystals of various sizes with no defined form. The MEO-GA decreased in particle size and changed in morphology compared to GA. The SEM micrographs of GA showed continuous tissues, and those of MEO-GA exhibited porous surfaces with amorphous shapes. Their porous surfaces were caused by the amount of essential oil presented in the GA.

**Figure 7:**
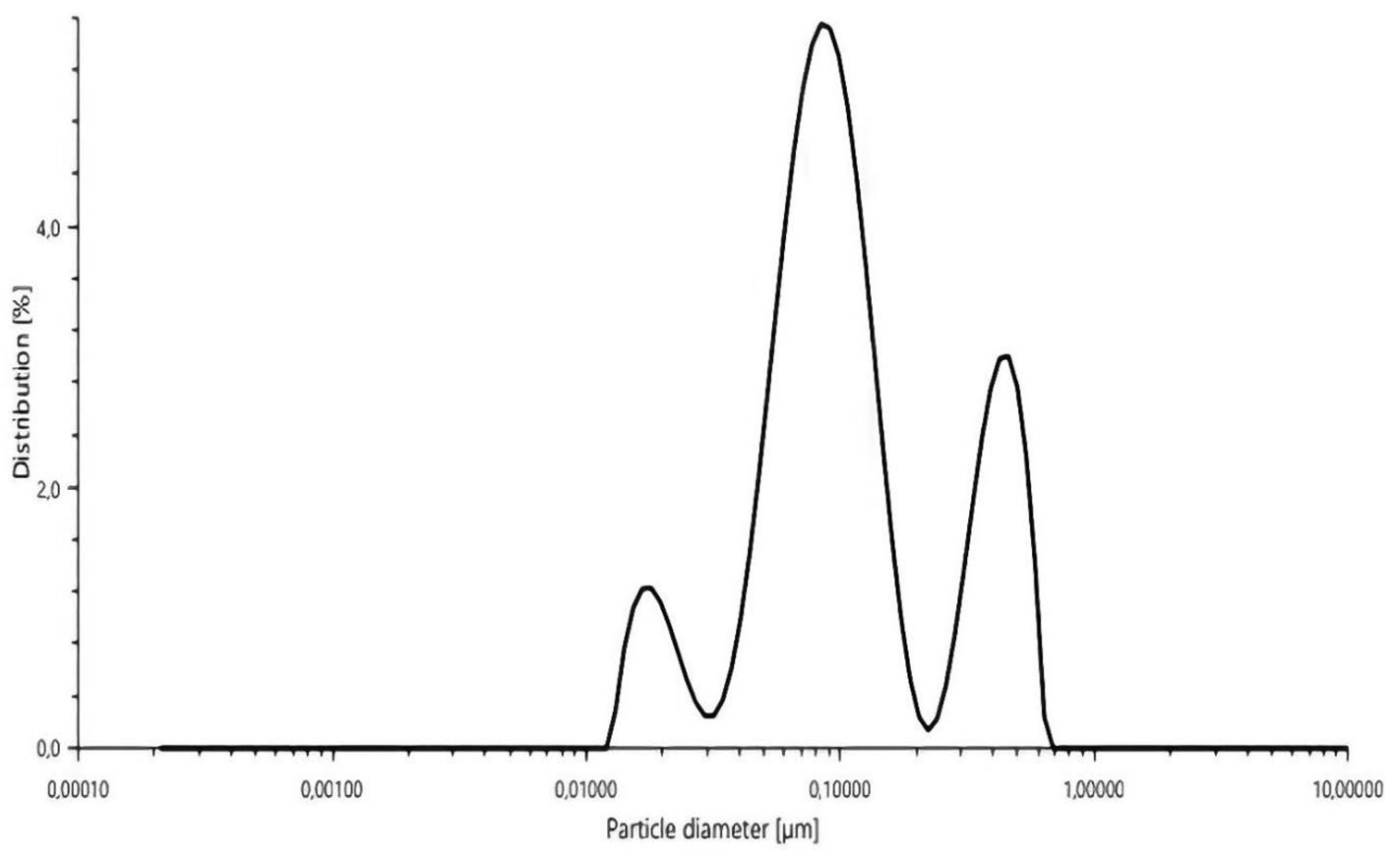
Particle size (µm) of MEO-GA nanoparticles distribution.

The mean hydrodynamic diameter of the nanoparticles was measured using dynamic light scattering (DLS). As shown in Fig. 7, the MEO-GA exhibited a mean diameter of 124.98 nm, which was consistent with the results attained by Matche and Adeogun^70^. These results seem to confirm previous research by Barre et al.^15^, showing that adding EO-to-GA decreased the particle size of the nanoencapsulated essential oil mixed with the GA. Hasheminejad et al.^71^ contended that this decrease may have been caused by the enhanced protonation of amino groups, leading to the completion of ionic cross-linking. Likewise, the nanoelaboration method’s reduction in nanocapsule particle size may be attributed to a greater packing of polymer chains because of the large number of amino groups in GA that induced EO–GA association^71^.

#### X-ray powder diffraction analysis

**Figure 8:**
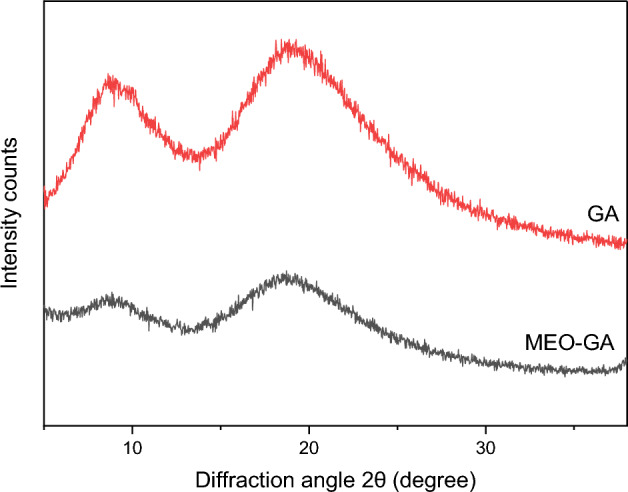
XRD patterns of GA and MEO-GA.

X-ray diffraction (XRD) was used to identify the differences in peaks’ positions and intensity in GA and MEO-GA. As shown in Fig. 8, the two samples were characterized by two large peaks at ~ 8° and 18° (2θ), with little modifications in position and significant variations in intensity. Other studies have also found that essential oil can successfully be incorporated into GA^72,73^.

#### Fourier transform infrared spectroscopy analysis

**Figure 9:**
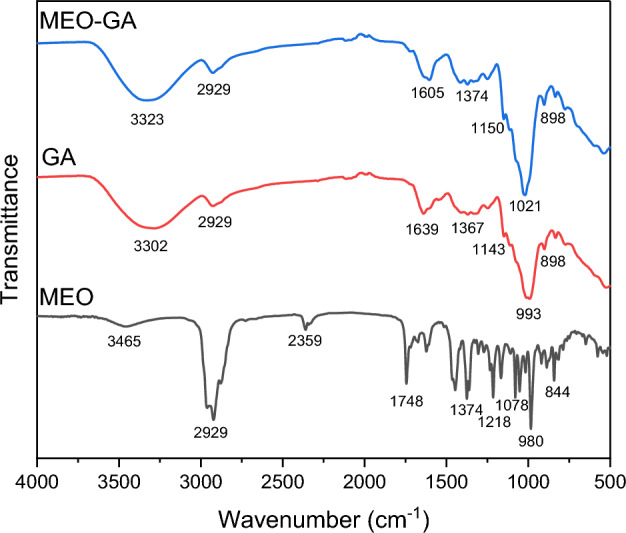
FTIR spectra of MEO, GA and MEO-GA.

Fourier transform infrared spectroscopy (FTIR) was performed to investigate the encapsulation of MOE in GA. The FTIR spectra of the MEO, GA, and MEO-GA samples are illustrated in Fig. 9. Differences in band position, shape, and intensity are indicators of interactions between the invited molecules and GA^74,75^. The MEO spectrum indicated that the characteristic broad band was at 3465 cm^−1^ relative to O–H bond stretching, and those of CH_2_ asymmetric stretching were at 2929 cm^−1^. The sharp band at 1748 cm^−1^ was assigned to the bending vibration of C=O groups, whereas the sharp bands at 1442 cm^−1^ and 1374 cm^−1^ were associated with CH_3_, CH_2_ and O–H bending, respectively. The bands at 1,218 cm^−1^, 1,078 cm^−1^, and 980 cm^−1^ were assigned to C–C and C–O bond stretching and to out-of-the-plane C–H and O–H stretching, respectively. These observations were consistent with the presence of 1,8-cineole, camphor, α-pinene, myrtenal, β-himachalene, and borneol, which were the main components of MEO (Table 3). Several authors have already studied the compounds presented in this essential oil^59,76^.

The spectra of GA was characterized by prominent vibrational peaks at 993 cm^−1^ (stretching vibrations of C-O), 1367 cm^−1^ (CH_3_ and C–H bending bands), 1639 cm^−1^ (C=O bond stretching), and 2929 cm^−1^ (CH_2_ asymmetric stretching). The band at 3302 cm^−1^ resulted from the hydrogen-bonded OH group^77^. The typical absorption band for the amino group of GA in the area of 3400–3500 cm^−1^ was likely obscured by the broad O–H group absorption band^33^.

The FTIR spectra of the MEO-GA formulations were a combination of previously characterized spectra. In addition, the C–H and O–H absorption bands were altered in position, intensity, and shape, thus indicating success in combining MEO with GA. The interaction of the polysaccharide with the essential oil may have influenced structural composition alteration^78^. According to Singh et al.^79^, the alterations generated by adding EO to GA indicated that chemical modifications occurred due to the incorporation of essential oils into GA. Consequently, the essential oil’s interaction with the functional groups contained in the polymer matrix led to identified modifications in peaks and displacements caused by stretching, vibrations, and bending. Furthermore, Vali et al.^80^ found that the peak shift indicated that the EO interacted with the GA due to the edible coatings and was effectively encapsulated.

#### Thermogravimetric analysis

**Figure 10:**
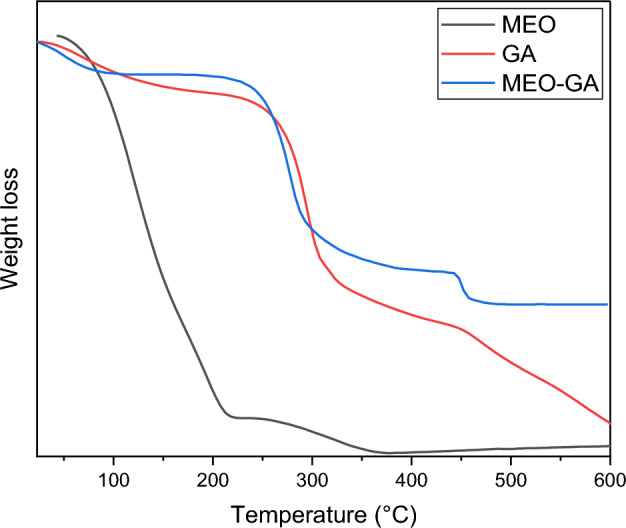
The TGA thermogram of MEO, GA and MEO-GA.

Thermogravimetric analysis (TGA) was demonstrated the thermal stability of essential oil after the encapsulation process^81^. The thermal behavior of EO was investigated in both forms (free and encapsulated) (Fig. 10). The MEO began to evaporate at room temperature, and the weight loss was 90% at 217 °C, illustrating MEO’s unstable nature. In the case of GA, the first one caused the dehydration process (112 °C), and the decomposition process (250 °C) caused the second one. In addition to GA’s dehydration and decomposition, the TGA of MEO-GA was characterized by additional weight loss caused by the volatilization of MEO until 440 °C. These findings provided further justification for the incorporation of MEO into GA and indicated that, during the encapsulation process, the MEO became more stable. The addition of MEO reduced the melting temperature of the nanoparticles, which could be related to the interaction of the EO with the polymer^82^. This illustrates the successful encapsulation of *S. rosmarinus* and *C. atlantica* (MEO) essential oil combined with GA and the bioactive compound’s encapsulation. This finding was comparable to that of Hadidi et al.^83^ study, which found that the incorporation formed between eucalyptus oil and GA led to the effective encapsulation of eucalyptus essential oil inside a carrier polymer (GA). Azadmanesh et al.^84^ determined that including *Eucalyptus globulus* oil in nanomaterials, such as GA nanocapsules, enhanced the nanocapsules’ thermal stability. Lian et al.^85^ established that contact between EO and polysaccharides could enhance the polysaccharide–EO complex’s thermal stability and that polysaccharides could improve heat resistance through intermolecular synergy, including hydrophobic synergy.

### Antifungal activity

The antifungal activity of the essential oil mixture, both in free and encapsulated forms, was among the most active forms examined. These samples were tested against two brown rot fungi (*P. placenta* and *G. trabeum*)*.*

**Figure 11:**
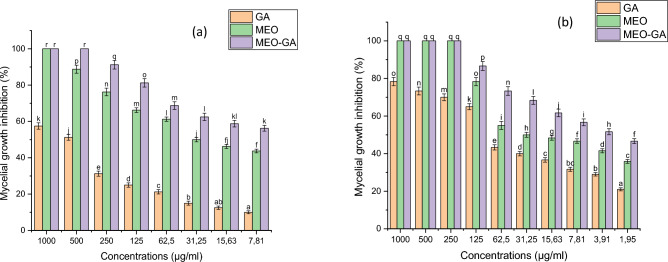
The inhibition percentages of GA, MEO, and MEO-GA against wood decay fungi growth of (**a**) *Poria placenta* and (**b**) *Gloeophyllum trabeum*.

**Figure 12:**
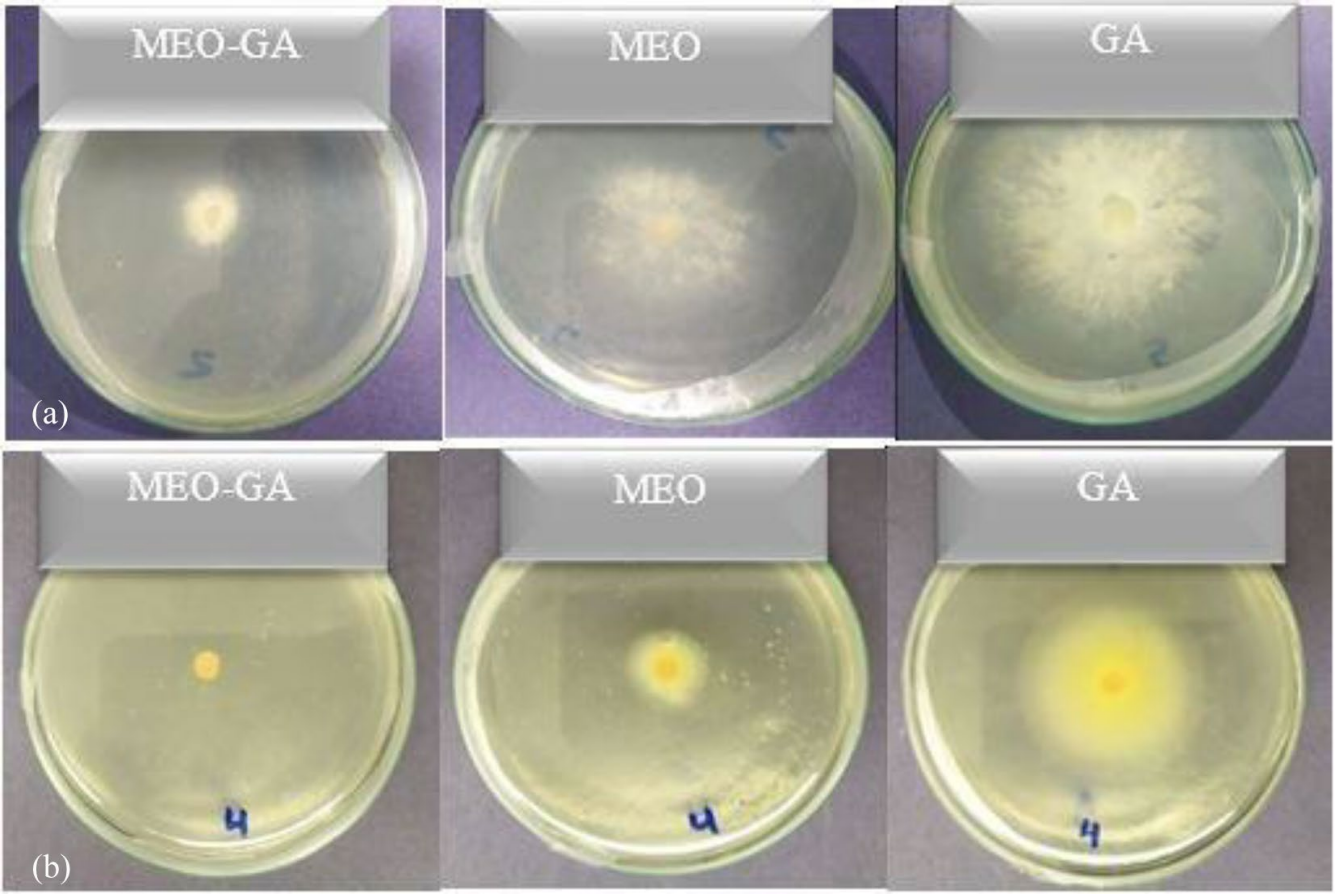
Antifungal activity of; essential oil-gum arabic (MEO-GA), mixture essential oil (MEO) and gum arabic (GA) against wood decay fungi growth of (**a**) *Poria placenta* and (**b**) *Gloeophyllum trabeum.* All in the concentration of 62.5 µg/mL. The negative control of two rot fungi on malt-agar medium was carried out by substituting the MEO-GA and GA with a 0.2% agar solution. After 7 days of incubation at 25 °C, the fungus fully covered the plates in the Petri dishes.

The percentage of mycelial growth inhibition in the essential oil mixture, both in free and encapsulated forms, against *P. placenta* and *G. trabeum* at different concentrations, was illustrated in Fig. 11. According to the results shown in Fig. 11, the percentage of mycelial growth inhibition increases with concentration, this response is dose-dependent, which all three graphs indicated. Figures 11 and 12 show significant differences between the samples in the average mycelial growth inhibition of *P. placenta* (Fig. 11a) and *G. trabeum* (Fig. 11b)*.* A significant difference (*p* < *0.05*) was identified in the average mycelial growth inhibition between (a) GA and MEO, (b) GA and MEO-GA, and (c) MEO and MEO-GA. The MEO-GA samples, followed by the MEO sample, showed the highest average mycelial growth inhibition for both brown rot fungi. The lowest average mycelial growth inhibition for both brown rot fungi was found in the GA samples. Based on two-way ANOVA tests, the applied treatment significantly affected mycelial growth inhibition for both brown rot fungi.

The effective concentration for 50% of mycelium growth (EC_50_) of the samples and chemical fungicide (Nystatin) are listed in Table 7, with a lower EC_50_ reflecting high antifungal activity. Table 7 indicates that MEO-GA provided the lowest EC_50_ values of all the fungi investigated (5.15 and 12.63 µg/mL for *P. placenta* and *G. trabeum, respectively*), followed by MEO (15.69 and 26.42 µg/mL for *P. placenta* and *G. trabeum, respectively*), and GA (144.88 and 327.36 µg/mL for *P. placenta* and *G. trabeum, respectively*).

**Table 7:** Effective concentration values (EC_50_) of GA, MEO-GA, and MEO against brown rot fungi.

Wood decay fungi	Samples	EC_50_ (µg/mL) (95% confidence intervals)^a^	Slope ± SE^b^	Intercept ± SE^c^	*R*^2^	*p*-value
*G. trabeum*	GA	144.88 (142.56–147.20)	0.83 ± 0.08	3.29 ± 0.19	0.97	0.00
	MEO-GA	5.15 (4.27–6.03)	1.22 ± 0.16	4.13 ± 0.34	0.95	0.00
	MEO	15.69 (14.89–16.49)	1.55 ± 0.24	3.12 ± 0.63	0.92	0.00
	Nystatin	6.71 (5.72–7.71)	1.82 ± 0.21	2.94 ± 0.59	0.94	0.00
*P. placenta*	GA	327.36 (324.15–330.57)	0.99 ± 0.12	2.48 ± 0.29	0.90	0.00
	MEO-GA	12.63 (11.98–13.28)	1.38 ± 0.19	3.48 ± 0.44	0.94	0.00
	MEO	26.42 (25.66–27.18)	1.41 ± 0.20	2.99 ± 0.49	0.92	0.00
	Nystatin	12.36 (11.51–13.22)	1.32 ± 0.16	3.48 ± 0.42	0.96	0.00

*GA* gum Arabic, *MEO* essential oil mixture, *MEO-GA* essential oil mixture encapsulated with Gum Arabic.

^a^Effective concentration for 50% of mycelium growth.

^b^Slope of regression line ± standard error.

^c^Intercept of regression line ± standard error.

Several researchers have investigated the application of GA in the nanoencapsulation of essential oils as food preservative^57^. Nevertheless, few studies have considered the application of encapsulated essential oils as wood protection against decay fungi^86^, and no studies have investigated the nanoencapsulation of a mixture of essential oils for application in wood protection. The strong antifungal effect of MEO could be attributed to the synergistic effect of the major compounds of *S. rosmarinus* (monoterpene) on those of *C. atlantica* (sesquiterpene), as Annemer et al.^22^ study indicated. MEO-GA, as an edible coating, showed more marked results for wood preservation than MEO against *P. placenta* and *G. trabeum.* This significant potential can be explained by synergistic actions in a mixture of essential oils with complex combinations of several components and GA. The authors Izadi et al.^87^, Ali et al.^21^, Cheong and Zahid^88^, Maqbool et al.^86^, and Valiathan and Athmaselvi^89^ observed that edible coatings, for instance, GA, could increase essential oil antifungal activity by attaching to the fungal surface and liberating their contents close to the cell membrane or transferring the fungal components immediately. In contrast, Maqbool et al.^86^ determined that GA molecules had no fungicidal effects. Lili Cai et al.^90^ indicated that four essential oils encapsulated with methyl-β-cyclodextrin (MβCD) substantially slowed the fungal growth of both fungi *P. placenta* and *G. trabeum*.

## Conclusion

In the present study, the nanoencapsulation of MEO in GA was optimized using a response surface design, and the effect of nanoencapsulation on antifungal activity was evaluated against brown rot fungi. As determined by characterization evaluation, the oil mixture was successfully nanoencapsulated into GA. The RSM results indicated that encapsulation efficiency could achieve a value of 87% in optimal conditions: 10% of MEO/ethanol and a 1:10 ratio of MEO/GA. The DLS data confirmed the nanoencapsulation process by measuring the average diameter. The SEM, FTIR and XRD analysis revealed that GA and MEO complexes exhibited different properties before and after the inclusion procedure, which enabled the conclusion of the incorporation process. The TGA results also indicated that the presented nanoparticles showed higher thermal stability than the MOE. Regarding antifungal activity, the nanoencapsulation of MEO with GA significantly improved the activity against *G. trabeum* and *P. placenta*. Therefore, the MEO-GA formulated in this research can be used as a green alternative for controlling brown rot fungi and, therefore, can potentially be applied in the wood industry.

## Data Availability

The data used to support the findings of this study are available from the corresponding author upon request.
